# Chemotaxis of Beneficial Rhizobacteria to Root Exudates: The First Step towards Root–Microbe Rhizosphere Interactions

**DOI:** 10.3390/ijms22136655

**Published:** 2021-06-22

**Authors:** Haichao Feng, Ruixin Fu, Xueqin Hou, Yu Lv, Nan Zhang, Yunpeng Liu, Zhihui Xu, Youzhi Miao, Tino Krell, Qirong Shen, Ruifu Zhang

**Affiliations:** 1 Jiangsu Provincial Key Lab for Organic Solid Waste Utilization, National Engineering Research Center for Organic-Based Fertilizers, Jiangsu Collaborative Innovation Center for Solid Organic Waste Resource Utilization, Nanjing Agricultural University, Nanjing 210095, China; hcfeng@njau.edu.cn (H.F.); 2016203021@njau.edu.cn (R.F.); 2019103141@njau.edu.cn (X.H.); 2020103140@Stu.Njau.Edu.Cn (Y.L.); nanzhang@njau.edu.cn (N.Z.); xzh2068@njau.edu.cn (Z.X.); yzmiao@njau.edu.cn (Y.M.); qirongshen@njau.edu.cn (Q.S.); 2 Key Laboratory of Microbial Resources Collection and Preservation, Ministry of Agriculture, Institute of Agricultural Resources and Regional Planning, Chinese Academy of Agricultural Sciences, Beijing 100081, China; liuyunpeng@caas.cn; 3 Department of Environmental Protection, Estación Experimental del Zaidín, Consejo Superior de Investigaciones Científicas, 18008 Granada, Spain; tino.krell@eez.csic.es

**Keywords:** rhizospheric chemotaxis, plant growth-promoting rhizobacteria (PGPR), methyl-accepting chemotaxis protein (MCP), dCache, chemoeffector

## Abstract

Chemotaxis, the ability of motile bacteria to direct their movement in gradients of attractants and repellents, plays an important role during the rhizosphere colonization by rhizobacteria. The rhizosphere is a unique niche for plant–microbe interactions. Root exudates are highly complex mixtures of chemoeffectors composed of hundreds of different compounds. Chemotaxis towards root exudates initiates rhizobacteria recruitment and the establishment of bacteria–root interactions. Over the last years, important progress has been made in the identification of root exudate components that play key roles in the colonization process, as well as in the identification of the cognate chemoreceptors. In the first part of this review, we summarized the roles of representative chemoeffectors that induce chemotaxis in typical rhizobacteria and discussed the structure and function of rhizobacterial chemoreceptors. In the second part we reviewed findings on how rhizobacterial chemotaxis and other root–microbe interactions promote the establishment of beneficial rhizobacteria-plant interactions leading to plant growth promotion and protection of plant health. In the last part we identified the existing gaps in the knowledge and discussed future research efforts that are necessary to close them.

## 1. Introduction

Plant-associated microbiomes, also referred to as the second genome of the plant [1], are crucial for plant health, such as growth promotion and disease resistance, etc. [2,3]. These microbiomes have formed a multifunction ‘holobiont’ with their plant host during evolution [4]. Therefore, the plant colonization by beneficial rhizobacteria (including *Bacillus*, *Pseudomonas*, *Azorhizobium*, etc.) is a process of enormous relevance for the establishment of sustainable and green agriculture production. Importantly, chemotaxis to seed or root exudates was shown to be an essential prerequisite for efficient root colonization. Plant-growth-promoting rhizobacteria (PGPRs), including members of many bacterial genera like *Bacillus*, *Pseudomonas*, *Azospirillum*, *Burkholderia*, and *Rhizobium* are widely used in agricultural production for stimulating plant growth and suppressing soil-borne diseases [5]. Further studies showed that the performance of their plant-beneficial effects depends on efficient rhizosphere colonization [6]. The rhizosphere, which is influenced by root exudates, can hold up to 10^11^ microbial cells per gram of root [7], that belong to more than 30,000 bacterial species [1]. To survive and thrive in the rhizosphere, rhizobacteria need to navigate in chemical gradients to places where their metabolism is optimal. Therefore, rhizosphere chemotaxis contributes to the overall structure of the microbial community in the rhizosphere.

Plant root exudates are a complex mixture of organic and inorganic substances released into the rhizosphere environment through the root system during plant growth [8]. Plants release up to 40% of their photosynthesis products into the rhizosphere as root exudates, that can be divided into two classes, namely small molecular weight compounds (including amino acids, organic acids, sugars, and other secondary metabolites), accounting for much of the diversity of root exudates, and the less diverse high molecular weight compounds, such as mucilage (polysaccharides) and proteins [8]. The root exudates of different plants are different in quantity and composition [9], depending on different factors, such as plant species [10], plant development [11], and soil environmental conditions [12]. However, numerous compounds are shared in different plants, and others rather unique to certain plants species have been identified. For example, benzoxazinoids (BXs), appear to be produced by different grass species (Poaceae, including maize, wheat, and rye) [13], mostly during the early stages of plant development and to a lesser extent in mature plants [14]. Many root exudate components serve as carbon and nitrogen sources for rhizosphere microoranisms and also play a role in the signaling processes that regulate the plant–bacteria interactions [15]. 

Plants recruit PGPRs to the rhizosphere through the release of specific signal molecules [16]. Chemotactic responses to root exudates, initiated by ligand sensing at chemoreceptors, or methyl-accepting chemotaxis proteins (MCPs), is very important for root colonization and the beneficial functions of PGPRs. It has been shown that chemotaxis in response to root exudates is an essential initial step in the recruitment and colonization of plants by PGPRs [17,18], that also enhances the ability of bacteria to colonize the roots of diverse plant hosts [18]. Given the complexity of root exudates, it is essential to identify components that play a key role in both chemotaxis and root colonization. 

Significant progress has been made in understanding rhizosphere chemotaxis, resulting in (i) the identification of chemoeffectors sensed by the rhizobacteria, (ii) the structural and functional characterization of a great diversity of rhizobacterial MCPs, and (iii) the determination of the influence of rhizosphere chemotaxis on root colonization, biofilm formation, and other root–microbe interactions. These advances will be summarised in this review and future research objectives are discussed in order to close the existing gaps of knowledge.

## 2. Chemoeffectors in Root Exudates Sensed by Beneficial Rhizobacteria

Root exudates contain primary and secondary metabolites, including amino acids, organic acids, sugars, alcohols, polyamines, fatty acids, purines, phytohormones, terpenoids, flavonoids, and benzoxazinoids [19,20]. So far, most of the identified chemoattractants of root exudates for rhizobacteria were low-molecular-weight compounds. Although plant root exudates are diverse and complex [8], primary metabolites are present in most of them. *Bacillus* spp. are important members of the PGPRs family, and have been commercially exploited as biofertilizers and biocontrol agents [21]. The well-studied and commercially widely used PGPR strain *Bacillus velezensis* SQR9, isolated from the cucumber rhizosphere, can promote plant growth, and protect plant health [22]. SQR9 is chemotactically attracted to cucumber root exudates [23]. The composition of cucumber root exudates was determined by mass spectrometry and it was shown that SQR9 responded chemotactically to nearly half of the tested root exudate components (44 of the 98 compounds). Of these 39 were chemoattractants (including 20 amino acids, 11 organic acids, 5 sugars, and 3 others), while only 5 compounds (salicylic acid, pentadecanoic acid, sodium decanoate, DL-dithiothreitol, and hydroxycarbamate) were chemorepellents [24]. 

Amino acids, organic acids, and sugars were the major chemoattractant families and accounted for 45, 32, and 11% of the identified chemoattractants (Table 1), respectively. *Pseudomonas putida* KT2440, an efficient colonizer of plant roots, can protect plant hosts against phytopathogens through the induction of systemic resistance. KT2440 was found to respond to many different organic acids (including TCA intermediates), amino acids, sugars, polyamines, gamma-aminobutyric acid (GABA), inorganic phosphate, and the changes in the energy status (Table 1) [25]. *Sinorhizobium* (*Ensifer*) *meliloti* is the cognate symbiont for alfalfa (*Medicago sativa* L.), which is a very important forage crop and capable of nitrogen-fixing symbiosis. Alfalfa and other legumes can recruit *S. meliloti* to the rhizosphere, exhibiting positive chemotactic responses toward a wide range of substances, such as amino acids, organic acids, sugars, and substrates that induce energy taxis (Table 1) [26]. 

However, as the composition of root exudates is complex, the resulting chemotactic behavior is highly multifactorial and represents the sum of activities of different chemoattractants and MCPs mediating responses with different magnitudes. Furthermore, in the rhizosphere, the concentration of root exudates varies with the different distance toward the root [42], and this variation in concentration adds to the complexity of chemotaxis and root colonization. 

Recent advances in untargeted metabolomics have enabled the detection and identification of a large number of novel exudate components [43]. Some compounds may be present in different forms, such as isomers or optical rotation, which may cause different chemotactic responses. For example, *Pseudomonas fluorescens* Pf0-1 showed a very strong response to L-malate, but could not sense D-malate [37]. A large unexplored field is the mutual influence of the tripartite probiotics-plant-pathogen interaction in root exudate chemoataxis and colonization. One such example is that of the cucumber root colonization by *Fusarium oxysporum f.sp.cucumerinum* that increased tryptophan secretion by the root, which is a strong chemoattractant of the PGPR strain *Bacillus velezensis* SQR9 leading to an enhanced root colonization that counteracts the effects of the pathogen [24,44]. 

In addition to the above common chemotactic compounds, there are some plant compounds that appear to be secreted specifically by some bacteria only [19,45,46]. In *S. meliloti*, quaternary ammonium compounds (QACs), such as glycine betaine, trigonelline, and choline, can serve as nutrient sources and osmoprotectants, and can promote the colonization of alfalfa seedling roots (Table 1). Like flavonoids, QACs (stachydrine and trigonelline) have been described to induce *nodD2* gene activity, which is critical in establishing the legume–rhizobium symbiosis [40,47]. Another example are isoflavones that are released by soybean roots and attract the nodulating symbiont *Bradyrizobium japonicum* [48]. Polyamines such as cadaverine, putrescine, and 1,3-diaminopropane, that are present in soybean root exudates, attract the plant parasite *Meloidogyne incognita* (a nematode). This knowledge permits the rational development of strategies for the protection of crops from the infection of root-knot nematodes [49]. The compound 1-aminocyclopropane-1-carboxylic acid (ACC), a precursor of ethylene synthesis in plants, is secreted in the rhizosphere, in which PGPRs can take advantage of it as a source of carbon and nitrogen using ACC deaminase, encoded by the *acdS* gene. ACC is a relatively strong and key chemoattractant for the strain *Pseudomonas* sp. UW4. Interestingly, most PGPRs contain the *acdS* gene, and can use ACC as the sole nitrogen source, giving them a significant advantage over competing bacteria [50].

The canonical way of sensing by MCPs consists in the interaction of the chemoeffector with the ligand binding domain (LBD). In recent years, a number of unconventional sensing mechanisms have been reported, such as those of pH and ethanol. Interestingly, *Bacillus subtilis*, that preferentially lives in environments with neutral pH, was found to perform bidirectional pH taxis. This response is based on the concerted action of four MCPs, namely McpA and TlpA for sensing acidic environments, and McpB and TlpB for sensing the alkaline pH range [51]. Ethanol is also an attractant for *B. subtilis*. This response appears to be counter-intuitive since ethanol is of no metabolic value to *B. subtilis* and, in addition, inhibits its growth. However, a possible reason for ethanol taxis may be to prey for ethanol-producing microorganisms [28]. These findings enlarge the list of known chemoeffectors.

Energy taxis relies on the activity of energy taxis receptors and is the movement to locations that permit optimal metabolic activities [52,53]. As one of the most important parameters, the redox state of the rhizosphere, is helpful for maintaining this ecological system [54,55]. Energy taxis to redox-active compounds may play major roles in plant–microbe interactions. A representative example is that of *Pseudomonas chlororaphis* that was isolated from avocado roots and that showed biocontrol activity towards the pathogen *Rosellinia necatrix*. The former organism produces the highly redox-active compound phenazine causing energy taxis involving multiple Aer receptors [56]. 

Furthermore, fungal hyphae can also release a series of compounds into the mycosphere, where they mediate fungi-bacteria interactions. The mycosphere is typically of weakly acidic, which favors the colonization by rhizobia, especially of the genus *Bradyrhizobium*. Since root exudate-driven bacterial chemotaxis cannot explain bacterial long-distance dispersal, mycelia constitute an ideal dispersal networks, which is also known as the “fungal highway”, that facilitates microbial transfer from bulk soil to the rhizosphere, which was found to be particularly important for establishing legume-rhizobium symbiosis [57]. Recently, spores of the nonmotile *Streptomyces* were found to activate chemotactic mechanisms of other soil bacteria (such as *Bacillus subtilis* and *Pseudomonas fluorescens*), which were transported from bulk soil to the plant roots. The authors suggested that *Streptomycetes* may be able to form “microbial hitchhiking” with their motile partner [58].

## 3. Rhizobacterial MCPs Sensing the Rhizosphere Chemoeffectors

The key component of a chemosensory pathway is the ternary complex formed by MCPs, the CheA histidine kinase, and the coupling protein CheW [59,60]. The canonical sensing mechanism consists in an interaction of the chemoeffector with the MCP LBD, that is typically located in the extracytoplasmic space. This interaction generates a molecular stimulus that is transduced across the membrane where it modulates CheA autokinase activity that consequently alters the transphosphorylation kinetics to the CheY response regulator [61]. The output of a chemosensory pathway is thus the modulation of the CheY to CheY-P ratio and only the latter is able to interact with the flagellar motor (FliM) causing ultimately chemotaxis [62].

Genome analysis revealed that bacteria contain on average of 14 MCP genes [63]. Furthermore, there is an enormous variety among MCPs in their topology and type of LBD they employ for sensing, since so far 80 different LBD types have been identified at MCPs [25]. MCPs that employ LBDs of the same class can recognize different ligands. On the contrary, a single ligand can also be recognized by structurally different LBDs [25,64]. The LBD families, Cache, 4HB, and PAS were found to be the most abundant among bacteria [25] and were shown to bind a very wide variety of signals. Studies have revealed that the abundance of MCPs is closely related to the bacterial lifestyle but not to its genome size [17]. Within the same species, the number of MCPs can vary significantly depending on their ecologial niche. For example, bacteria that inhabit aquatic and soil environments possess more MCPs, while species that are usually found in specific, confined, and relatively constant environments, have fewer MCPs [25,65]. Most soil bacteria can perform chemotaxis, and in general, chemotactic genes are enriched in bacteria from the rhizosphere as compared to those of the bulk soil [18]. Bacteria containing more MCPs also typically possess complex behaviors, such as the ability to establish relationships with other organisms. For example, strains of legume symbiont *Rhizobium leguminosarum* and the plant tumor causing *Agrobacterium tumefaciens* possess 20–60 *mcp* genes per genome [66]. 

A large number of MCPs and their corresponding ligands have been characterized in different rhizobacteria, including *Pseudomonas putida*, *Bacillus subtilis* and *Sinorhizobium meliloti* [29,40,67,68]. More than 140 compounds have been found to induce chemotaxis in *Pseudomonas* strains [69], which made these strains models for establishing MCP structure-function relationships [70]. Twenty-seven MCPs from the free-living environmental strain *P. putida* KT2440 have been characterized, of which McpA, McpG, McpH, McpP, McpQ, McpS, McpU, and PcaY_PP were identified as MCPs for amino acids (glycine, alanine, cystine, serine, asparagine, glutamine, phenylalanine, tyrosine, valine, isoleucine, methionine, arginine), GABA, purine (adenine and guanine), C2 and C3 carboxylic acids (acetate, pyruvate, propionate, and L-lactate), citrate/ metal ion complexes, tricarboxylic acid cycle intermediates (malate, fumarate, oxaloacetate, succinate, citrate, isocitrate, butyrate, acetate), polyamines (putrescine, cadaverine, and spermidine), and histamine as well as C6-ring containing carboxylic acids, respectively [31,32,34,69,71]. However, a large number of *P. putida* MCPs, remain functionally uncharacterized. The Gram-positive model bacterium *B. subtilis* OI1085 possesses 10 MCPs, namely McpA, McpB, McpC, TlpA, TlpB, TlpC, HemAT, YfmS, YvaQ, and YoaH [17]. McpC binds 11 amino acids directly (proline, threonine, glycine, serine, valine, alanine, tyrosine, isoleucine, phenylalanine, leucine, and tryptophan) and further evidence indicates that it may bind four others (lysine, arginine, methionine, and glutamine) using indirect binding mechanisms [29]. McpC was identified as an important MCP for rhizosphere chemotaxis and root colonization in *Arabidopsis thaliana* [17,29,72]. The chemotactic response of the alfalfa symbiont *Sinorhizobium meliloti* is mediated by nine different MCPs, including seven transmembrane MCPs (McpS to McpX and McpZ) and two soluble cytosolic receptors (McpY and IcpA), of which McpU and McpX play central roles in mediating host interactions by sensing plant-derived amino acids and QACs, respectively. McpV, the most abundant MCP in *S. meliloti*, accounts for 70% of the total MCP pool, senses short-chain carboxylates (including propionate, acetate, glycolate, pyruvate, acetoacetate, formate) via direct binding [26,38,40,73]. However, the function of the remaining six MCPs are unknown. 

Homologous MCPs in different strains may have different functions, as exemplified by McpA in *B. velezensis* SQR9 and *B. subtilis* NCIB 3610. McpA of *B. velezensis* SQR9 senses nine different dicarboxylic acids including two amino acids (aspartic acid and glutamic acid) and seven organic acids (citric acid, malic acid, oxalic acid, fumaric acid, succinic acid, phthalic acid, and adipic acid) [24]. In contrast, the McpA homolog of *B. subtilis* NCIB 3610 mediated attraction toward glucose and α-methylglucoside and might sense repellent molecule(s) secreted by *Arabidopsis thaliana* [17]. McpB, McpC, and TlpC mediated chemotaxis of the plant-associated strain *Bacillus subtilis* NCIB 3610 to *Arabidopsis thaliana* root exudates that was found to be required for early root colonization [17]. The oxygen sensing MCP IcpB of *Azorhizobium caulinodans*, which belong to a plant beneficial bacterium, had an influence on the nodulation and nitrogen fixation on the stems and roots of *Sesbania rostrata* [74]. The McpU of *Sinorhizobium meliloti*, mediated chemotaxis to proline and other plant-derived amino acids and plays an important role in chemotaxis to root exudates and rhizosphere colonization [38,40]. 

Previous studies of MCP transcript levels in the presence of different root exudate concentrations have revealed an increase in MCP expression at lower exudate concentrations (at a distance to the root), but a reduction at higher concentrations (in root vicinity). A model was proposed in which bacteria induce the chemosensory system at low exudate concentrations but inhibit this sytem at higher concentrations, in other words, when bacteria are close to the root, the chemotaxis is no longer required [75].

The role of the eight *B. velezensis* SQR9 MCPs in root exudate chemotaxis and cucumber root colonization has been established. A mutant that lacked all MCP genes was complemented with each of the individual MCPs. Interestingly only two MCPs, McpA, and McpC, participated in root exudate chemotaxis to an extent that the mutant strain complemented with both MCP genes showed wild type-like chemotaxis to root exudates. Very similar observations were made when the capacity of these eight MCPs in root colonization were assessed. In analogy to the above studies, only McpA and McpC were involved in root colonization and the strain harboring both genes showed wild type-like colonization phenotype [24,76]. This example clearly illustrates the crucial role of chemotaxis in the process of root colonization. The chemoeffectors that are sensed by both MCPs have been identified. Whereas McpA responds to malic acid, fumaric acid, glyceric acid, lysine, and mannose, McpC is stimulated by serine, alanine, and gluconic acid [76].

The dCache domain, composed of two α/β structural modules and a long N-terminal-helix, is one of the two characterized LBDs with a bimodular arrangement. In recent years, the dCache domain, as one of the most abundant LBD in MCPs, has become the focus in the study on bacterial chemotaxis. A significant number of dCache containing MCPs were characterized and found to bind a broad range of ligands. For example, *P. aeruginosa* PctA was identified to directly bind 17 amino acids and the autoinducer-2 (AI-2) [77,78]. In addition, PctA also mediates histamine chemotaxis using a mechanism that may involve indirect ligand recognition by solute binding protein [35,79].

Strikingly, a large number of dCache domain containing MCPs were found to participate in the chemotaxis to root exudates that are listed in Table 1. For example, *B. subtilis* OI1085 McpC is responsible for chemotaxis to all proteinogenic amino acids except L-asparagine and was found to bind 12 of them directly whereas the remianing ligands may also be recognized via solute binding proteins [29]. McpC is involved in *B. subtilis* chemotaxis to *Arabidopsis thaliana* root exudates, which was required for efficient root colonization [17]. *S. meliloti* McpU is involved in sensing amino acids, including all nonacidic proteogenic amino acids and several nonproteogenic amino acids [38]. McpX in the alfalfa symbiont *S. meliloti* governed chemotaxis towards host plant root exudates and participates in root colonization through directly QACs sensing [40,73]. The broadest ligand range of dCache LBD containg MCPs involved in root exudate chemotaxis is McpA of *B. velezensis* SQR9 that responds to 5 amino acids (aspartic acid, glutamic acid, isoleucine, lysine, and tyrosine), 10 organic acids (citric, malic, oxalic, fumaric, succinic, phthalic, adipic, dehydroascorbic, glyceric, and 3-hydroxypropionic acids), and 6 other compounds (hydroxycarbamate, mannose, ribose, fucose, galactose, and ribitol) [24,76,80]. These studies have shown that MCPs with dCache domain in rhizobacteria can sense many structurally different chemoeffectors.

**Figure 1:**
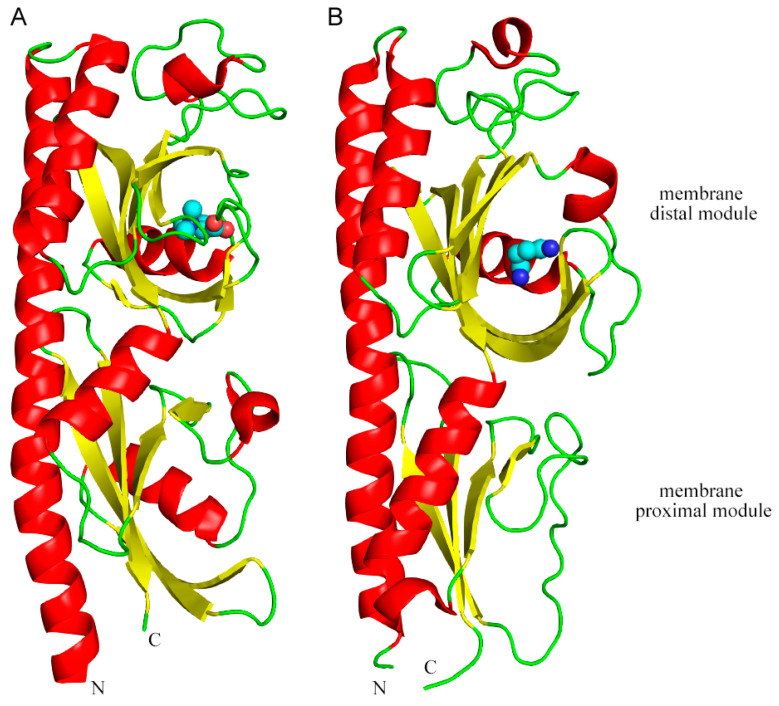
MCP of dCache sensor domain of rhizobacteria. Shown are the 3D structures of the dCache sensor domains of the *S. meliloti* McpX in complex with proline betaine (**A**) and the *P. putida* McpU in complex with putrescine (**B**). Bound ligands are shown in spheres mode. These structures have been published and are deposited in the protein databank with ID 6D8V [41] and 6F9G [34], respectively.

Structural and functional studies of dCache domains showed that ligands were almost exclusively bound to the membrane-distal module (Figure 1) [25,34,35,81,82], whereas there is only a single case for signal recognition at the membrane proximal module [83]. It was thus suggested that the primary roles of the membrane proximal module consists in the stimulus transmission to the transmembrane region [25] or the recognition of solute binding proteins [79]. A recent study demonstrated that the dCache domain containing McpB of *Bacillus subtilis* can directly bind ethanol at its cytoplasmic signaling domain, indicating the existance of alternative mechanisms for MCP stimulation [28]. In summary, dCache domain-containing MCPs recognize a variety of different compounds, such as proteinogenic amino acids, short-chain carboxylic acids, sugars, GABA, quaternary amines, purines, histamine and polyamines, taurine, and AI-2, and recognition occurs primarily at the membrane distal module. 

Since members of the same sensor domain family bind many different ligands, efforts have been made to identify specific features within the ligand binding pocket that determine the nature of the ligand recognized. This knowledge can then be used to predict ligands that bind to other uncharacterized MCPs. Comparative sequence analyses combined with the 3D strcutural information of the dCache sensor domains of the three paralogous MCPs PctA, PctB, and PctC of *P. aeruginosa* has led to the identification of a highly conserved amino acid recognition motif (Tyr121, Asp122, Arg124, Arg126, and Trp128) [84]. This motif was identified in many dCache domains that were previously identified to bind amino acids, but was absent from dCache domains that bind other ligands like polyamines, purines, or quaternary amines [76]. dCache domains that contain the amino acid binding motif show a wide phylogenetic distribution and have been identified in *Pseudomonas*, *Bacillus*, and *Sinorhizobium*. Further research will undoubtedly identify other ligands that are recognized by dCache domains and that are relevant in the context of root exudate chemotaxis. 

## 4. Role of Chemotaxis in Root–Microbe Interactions in Rhizosphere

Chemotaxis plays very important roles in root–microbe interactions in the rhizosphere—such as rhizoplane biofilm formation, root colonization, nitrogen fixation, and pathogenesis [17,30,85,86,87,88]—and a diverse range of MCPs were found to be involved in these interactions. Recently, we have shown that root-secreted D-galactose is an inducible signal that regulates chemotaxis and biofilm formation in the plant beneficial rhizobacteria SQR9 in an McpA-dependent manner [80]. In *P. putida*, mutants of either McpU, which is responsible for chemotaxis to polyamine, or WspA, which is another chemosensory pathway, were much less competitive than wild type for maize root colonization [30]. In *Rhizobium leguminosarum*, mutants of McpB and McpC are unable to compete with wild type cells in nodulation experiments of Trapper peas [89]. In *Azorhizobium caulinodans*, the oxygen sensing MCP IcpB modulates nodulation and nitrogen fixation on the stems and roots of *Sesbania rostrata*, and also affects the production of extracellular polysaccharides and impairs flocculation [74]. In pathogenic *Ralstonia pseudosolanacearum*, McpM-mediated chemotaxis to L-malate, secreted by tomato roots, is essential for the infection process [88]. All these studies demonstrate the importance of rhizosphere chemotaxis. 

## 5. Conclusions and Prospects 

**Figure 2:**
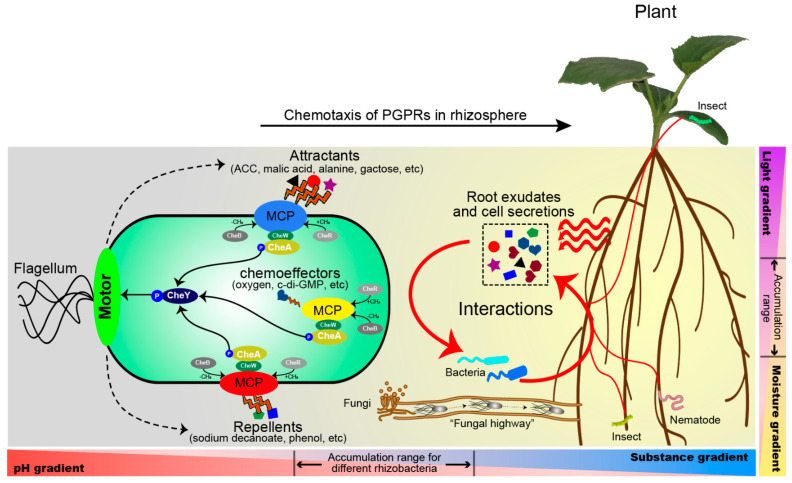
Model of rhizobacteria recruitment to plant roots through chemotaxis. Chemotaxis to plant roots is a prerequisite for efficient root colonization by PGPRs, which plays important roles for plant growth and health. Roots produce chemical compounds that attract beneficial bacteria and repel harmful bacteria. The composition of root exudates is influenced by a variety of factors, such as biotic and abiotic factors. In addition to root exudates, soil microorganisms in the rhizosphere can also produce some secretions, thereby affecting the movement of rhizobacteria, such as the interaction between rhizobia and filamentous fungi. Mycelia may constitute an ideal dispersal networks, known as “fungal highway” for the rhizobial long-distance dispersal, to promote rhizobial enrichment in the legume rhizosphere from bulk soil. Otherwise, the rhizosphere environment is also influenced by many factors such as light, moisture, pH, redox, etc., which may be helpful for rhizobacteria to move toward to rhizosphere.

A large number of in-depth and systematic studies have led to the functional annotation of MCPs with their chemoeffectors in different flagellated bacteria. We have tried to summarize the resulting knowledge in Figure 2, which suggests that amino acid and organic acid chemotaxis is of particular relevance for root exudate chemotaxis. In addition, a number of chemorepellents have been identified, but their physiological relevance still remains to be established. The study of rhizosphere chemotaxis forms the basis for biotechnological applications aimed at improving plant colonization by PGPRs using genetic engineering approaches. One possible strategy for reinforcing root exudate chemotaxis is to increase the cellular abundance of the key MCPs in plant beneficial rhizobacteria or, alternatively, the stimulation of the exudation of dominant attractants by host plants. 

An interesting research of the rhizosphere chemotaxis is to mimic the in situ process. However, due to the complex interactions in rhizosphere and the diversity of rhizomicrobiome, it will be a big challenge to clarity the molecular mechanism of in situ rhizosphere chemotaxis, that is also meaningful and helpful to explain how plants recruit rhizosphere microorganisms.

While insightful, many of these studies only rely on the relatively constant and confined laboratory conditions to elucidate the roles of MCPs in sensing specific compounds within root exudates and plant colonization. Future studies are necessary to elucidate the role of chemotaxis and in particular (i) to understand the structural basis for signal recognition at broad ligand-range MCPs; (ii) to identify the key chemoattractants sensed by different beneficial rhizobacteria in the rhizosphere of different crops; (iii) to elucidate the role of chemotaxis in the definition of the rhizosphere microbiome; (iv) to exploit the gained knowledge on rhizosphere chemotaxis to engineer bacteria and plants as a strategy to contribute to the establishment of a sustainable agriculture.

## Figures and Tables

**Table 1:** MCPs of rhizobacteria containing a cache type sensor domain that respond to different ligands.

Strains (Total Number of MCPs)	MCP	Chemoeffector	Binding Mode	References
*Bacillus subtilis* OI1085 (10)	McpA	Glucose, alpha-D-ethylglucoside	unknown	[27]
	McpB	Asparagine, aspartic acid, glutamine, histidine; methanol	direct	[27,28]
	McpC	Proline, threonine, glycine, serine, valine, alanine, tyrosine, isoleucine, tryptophan, phenylalanine, leucine	direct	[29]
		Asparagine, lysine, glutamine, methionine	indirect *	
*Bacillus amyloliquefaciens* SQR9 (8)	McpA	Citric acid, aspartic acid	direct	[24]
		Glutamic acid, isoleucine, lysine, tyrosine, serine; phthalic acid, oxalic acid, malic acid, succinic acid, fumaric acid, adipic acid, dehydroascorbic acid, glyceric acid, 3-hydroxypropionic acid, gluconic acid, sodium decanoate; ribitol, mannose, ribose, fucose, hydroxycarbamate ^(r)^, fructose, galactose	unknown	
	McpB	Glycine, tryptophan, asparagine, glutamine, serine, cysteine, methionine; salicylic acid ^(r)^, sodium decanoate ^(r)^, adipic acid, ribose, glyceric acid, 3-hydroxypropionic acid, gluconic acid, fructose	unknown	
	McpC	Valine, alanine, proline, leucine, histidine, serine, threonine, cysteine, methionine; gluconic acid, succinic acid, maltose	unknown	
	TlpA	DL-dithiothreitol ^(r)^, hydroxycarbamate ^(r)^, sodium decanoate ^(r)^, gluconic acid, maltose	unknown	
	TlpB	Phenylalanine, threonine; malic acid, succinic acid, fumaric acid, salicylic acid ^(r)^, sodium decanoate ^(r)^, gluconic acid, pentadecanoic acid ^(r)^; ribose, fucose, maltose, fructose; dulcitol, inosine	unknown	
*Pseudomonas putida* KT2440 (27)	McpA	Glycine, alanine, cysteine, serine, asparagine, glutamine, phenylalanine, tyrosine, valine, isoleucine, methionine, arginine	direct	[30]
	McpG	γ-aminobutyric acid (GABA)	direct	[31]
	McpH	Adenine, guanine, hypoxanthine, purine, xanthine, uric acid	direct	[32]
	McpP ^(s)^	Acetate, pyruvate, propionate, L-lactate	direct	[33]
	McpU	Agmatine, cadaverine, ethylenediamine, histamine, putrescine, spermidine	direct	[30,34,35]
*Pseudomonas fluorescens* Pf0-1 (37)	CtaA	Alanine, arginine, asparagine, cystine, glycine, histidine, isoleucine, lysine, methionine, phenylalanine, serine, threonine, tyrosine, valine, leucine, proline	unknown	[36]
	CtaB	Alanine, arginine, asparagine, cystine, glycine, histidine, isoleucine, lysine, methionine, phenylalanine, serine, threonine, tyrosine, valine, glutamine, glutamic acid	unknown	
	CtaC	Arginine, cystine, glycine, methionine, threonine	unknown	
	McpT ^(s)^	L-malate, succinate	unknown	[37]
*Sinorhizobium meliloti* MV II-1 (8)	McpU	Proline	direct	[38,39]
	McpV ^(s)^	Propionate, acetate, glycolate, pyruvate, acetoacetate, formate	direct	[26,39]
		Butyrate, L-lactate, glyoxylate, methyl pyruvate, α-hydroxybutyrate, α-ketobutyrate;	unknown	
	McpX	Choline, glycine betaine, stachydrine, trigonelline, choline, betonicine, proline betaine	direct	[39,40,41]

^(r)^ indicates that this compound is a chemorepellent. ^(s)^ indicates that the LBD of the MCP belongs to sCache domain family, whereas the remaining MCPs possess a dCache domain. * indicates that three candidate binding lipoproteins associated with amino acid transporters: ArtP was found to bind arginine and lysine; GlnH, glutamine; MetQ, methionine.

## References

[1] Mendes R., Kruijt M., de Bruijn I., Dekkers E., van der Voort M., Schneider J.H., Piceno Y.M., DeSantis T.Z., Andersen G.L., Bakker P.A. (2011). Deciphering the Rhizosphere Microbiome for Disease-Suppressive Bacteria. Science.

[2] De Vries F.T., Griffiths R.I., Knight C.G., Nicolitch O., Williams A. (2020). Harnessing Rhizosphere Microbiomes for Drought-Resilient Crop Production. Science.

[3] Trivedi P., Leach J.E., Tringe S.G., Sa T., Singh B.K. (2020). Plant–Microbiome Interactions: From Community Assembly to Plant Health. Nat. Rev. Microbiol..

[4] Vandenkoornhuyse P., Quaiser A., Duhamel M., Van Le A., Dufresne A. (2015). The Importance of the Microbiome of the Plant Holobiont. New Phytol..

[5] Lugtenberg B., Kamilova F. (2009). Plant-Growth-Promoting Rhizobacteria. Annu. Rev. Microbiol..

[6] Compant S., Clément C., Sessitsch A. (2010). Plant Growth-Promoting Bacteria in the Rhizo- and Endosphere of Plants: Their Role, Colonization, Mechanisms Involved and Prospects for Utilization. Soil Biol. Biochem..

[7] Egamberdieva D., Kamilova F., Validov S., Gafurova L., Kucharova Z., Lugtenberg B. (2008). High Incidence of Plant Growth-Stimulating Bacteria Associated with the Rhizosphere of Wheat Grown on Salinated Soil in Uzbekistan. Environ. Microbiol..

[8] Bais H.P., Weir T.L., Perry L.G., Gilroy S., Vivanco J.M. (2006). The Role of Root Exudates in Rhizosphere Interactions with Plants and Other Organisms. Annu. Rev. Plant Biol..

[9] Kong H.G., Song G.C., Sim H.-J., Ryu C.-M. (2021). Achieving Similar Root Microbiota Composition in Neighbouring Plants through Airborne Signalling. ISME J..

[10] Mönchgesang S., Strehmel N., Schmidt S., Westphal L., Taruttis F., Müller E., Herklotz S., Neumann S., Scheel D. (2016). Natural Variation of Root Exudates in *Arabidopsis thaliana*-Linking Metabolomic and Genomic Data. Sci. Rep..

[11] Chaparro J.M., Badri D.V., Vivanco J.M. (2014). Rhizosphere Microbiome Assemblage Is Affected by Plant Development. ISME J..

[12] Carvalhais L.C., Dennis P.G., Fan B., Fedoseyenko D., Kierul K., Becker A., von Wiren N., Borriss R. (2013). Linking Plant Nutritional Status to Plant–Microbe Interactions. PLoS ONE.

[13] Frey M., Schullehner K., Dick R., Fiesselmann A., Gierl A. (2009). Benzoxazinoid Biosynthesis, a Model for Evolution of Secondary Metabolic Pathways in Plants. Phytochemistry.

[14] Kudjordjie E.N., Sapkota R., Steffensen S.K., Fomsgaard I.S., Nicolaisen M. (2019). Maize Synthesized Benzoxazinoids Affect the Host Associated Microbiome. Microbiome.

[15] Chagas F.O., Pessotti R.D.C., Caraballo-Rodríguez A.M., Pupo M.T. (2018). Chemical Signaling Involved in Plant–Microbe Interactions. Chem. Soc. Rev..

[16] Bardy S.L., Briegel A., Rainville S., Krell T. (2017). Recent Advances and Future Prospects in Bacterial and Archaeal Locomotion and Signal Transduction. J. Bacteriol..

[17] Allard-Massicotte R., Tessier L., Lecuyer F., Lakshmanan V., Lucier J.F., Garneau D., Caudwell L., Vlamakis H., Bais H.P., Beauregard P.B. (2016). *Bacillus subtilis* Early Colonization of *Arabidopsis thaliana* Roots Involves Multiple Chemotaxis Receptors. mBio.

[18] Scharf B.E., Hynes M.F., Alexandre G.M. (2016). Chemotaxis Signaling Systems in Model Beneficial Plant–Bacteria Associations. Plant Mol. Biol..

[19] Badri D.V., Vivanco J.M. (2009). Regulation and Function of Root Exudates. Plant. Cell Environ..

[20] Pétriacq P., Williams A., Cotton A., McFarlane A.E., Rolfe S.A., Ton J. (2017). Metabolite Profiling of Non-Sterile Rhizosphere Soil. Plant J..

[21] Pérez-García A., Romero D., de Vicente A. (2011). Plant Protection and Growth Stimulation by Microorganisms: Biotechnological Applications of Bacilli in Agriculture. Curr. Opin. Biotechnol..

[22] Cao Y., Zhang Z., Ling N., Yuan Y., Zheng X., Shen B., Shen Q. (2011). *Bacillus subtilis* SQR9 Can Control Fusarium Wilt in Cucumber by Colonizing Plant Roots. Biol. Fertil. Soils.

[23] Zhang N., Wang D., Liu Y., Li S., Shen Q., Zhang R. (2013). Effects of Different Plant Root Exudates and Their Organic Acid Components on Chemotaxis, Biofilm Formation and Colonization by Beneficial Rhizosphere-Associated Bacterial Strains. Plant Soil.

[24] Feng H., Zhang N., Du W., Zhang H., Liu Y., Fu R., Shao J., Zhang G., Shen Q.R., Zhang R. (2018). Identification of Chemotaxis Compounds in Root Exudates and Their Sensing Chemoreceptors in Plant Growth-Promoting Rhizobacteria *Bacillus amyloliquefaciens* SQR9. Mol. Plant Microbe Interact..

[25] Ortega Á., Zhulin I.B., Krell T. (2017). Sensory Repertoire of Bacterial Chemoreceptors. Microbiol. Mol. Biol. Rev..

[26] Compton K.K., Hildreth S.B., Helm R.F., Scharf B.E. (2018). *Sinorhizobium meliloti* Chemoreceptor McpV Senses Short-Chain Carboxylates via Direct Binding. J. Bacteriol..

[27] Hanlon D.W., Ordal G.W. (1994). Cloning and Characterization of Genes Encoding Methyl-Accepting Chemotaxis Proteins in *Bacillus subtilis*. J. Biol. Chem..

[28] Tohidifar P., Bodhankar G.A., Pei S., Cassidy C.K., Walukiewicz H.E., Ordal G.W., Stansfeld P.J., Rao C.V. (2020). The Unconventional Cytoplasmic Sensing Mechanism for Ethanol Chemotaxis in *Bacillus subtilis*. mBio.

[29] Glekas G.D., Mulhern B.J., Kroc A., Duelfer K.A., Lei V., Rao C.V., Ordal G.W. (2012). The *Bacillus subtilis* Chemoreceptor McpC Senses Multiple Ligands Using Two Discrete Mechanisms. J. Biol. Chem..

[30] Corral-Lugo A., De la Torre J., Matilla M.A., Fernández M., Morel B., Espinosa-Urgel M., Krell T. (2016). Assessment of the Contribution of Chemoreceptor-Based Signalling to Biofilm Formation. Environ. Microbiol..

[31] Reyes-Darias J.A., García V., Rico-Jiménez M., Corral-Lugo A., Lesouhaitier O., Juárez-Hernández D., Yang Y., Bi S., Feuilloley M., Muñoz-Rojas J. (2015). Specific Gamma-Aminobutyrate Chemotaxis in Pseudomonads with Different Lifestyle. Mol. Microbiol..

[32] Fernández M., Morel B., Corral-Lugo A., Krell T. (2016). Identification of a Chemoreceptor That Specifically Mediates Chemotaxis toward Metabolizable Purine Derivatives. Mol. Microbiol..

[33] Garcia V., Reyes-Darias J.A., Martin-Mora D., Morel B., Matilla M.A., Krell T. (2015). Identification of a Chemoreceptor for C2 and C3 Carboxylic Acids. Appl. Environ. Microbiol..

[34] Gavira J.A., Ortega Á., Martín-Mora D., Conejero-Muriel M.T., Corral-Lugo A., Morel B., Matilla M.A., Krell T. (2018). Structural Basis for Polyamine Binding at the DCACHE Domain of the McpU Chemoreceptor from *Pseudomonas putida*. J. Mol. Biol..

[35] Corral-Lugo A., Matilla M.A., Martín-Mora D., Silva Jiménez H., Mesa Torres N., Kato J., Hida A., Oku S., Conejero-Muriel M., Gavira J.A. (2018). High-Affinity Chemotaxis to Histamine Mediated by the TlpQ Chemoreceptor of the Human Pathogen *Pseudomonas aeruginosa*. mBio.

[36] Oku S., Komatsu A., Tajima T., Nakashimada Y., Kato J. (2012). Identification of Chemotaxis Sensory Proteins for Amino Acids in *Pseudomonas fluorescens* Pf0-1 and Their Involvement in Chemotaxis to Tomato Root Exudate and Root Colonization. Microbes Environ..

[37] Oku S., Komatsu A., Nakashimada Y., Tajima T., Kato J. (2014). Identification of *Pseudomonas fluorescens* Chemotaxis Sensory Proteins for Malate, Succinate, and Fumarate, and Their Involvement in Root Colonization. Microbes Environ..

[38] Webb B.A., Helm R.F., Scharf B.E. (2016). Contribution of Individual Chemoreceptors to *Sinorhizobium meliloti* Chemotaxis Towards Amino Acids of Host and Nonhost Seed Exudates. Mol. Plant Microbe Interact..

[39] Meier V.M., Muschler P., Scharf B.E. (2007). Functional Analysis of Nine Putative Chemoreceptor Proteins in *Sinorhizobium meliloti*. J. Bacteriol..

[40] Webb B.A., Karl Compton K., Castaneda Saldana R., Arapov T.D., Keith Ray W., Helm R.F., Scharf B.E. (2017). *Sinorhizobium meliloti* Chemotaxis to Quaternary Ammonium Compounds Is Mediated by the Chemoreceptor McpX. Mol. Microbiol..

[41] Shrestha M., Compton K.K., Mancl J.M., Webb B.A., Brown A.M., Scharf B.E., Schubot F.D. (2018). Structure of the Sensory Domain of McpX from *Sinorhizobium meliloti*, the First Known Bacterial Chemotactic Sensor for Quaternary Ammonium Compounds. Biochem. J..

[42] Sasse J., Martinoia E., Northen T. (2018). Feed Your Friends: Do Plant Exudates Shape the Root Microbiome?. Trends Plant Sci..

[43] van Dam N.M., Bouwmeester H.J. (2016). Metabolomics in the Rhizosphere: Tapping into Belowground Chemical Communication. Trends Plant Sci..

[44] Liu Y., Chen L., Wu G., Feng H., Zhang G., Shen Q., Zhang R. (2017). Identification of Root-Secreted Compounds Involved in the Communication Between Cucumber, the Beneficial *Bacillus amyloliquefaciens*, and the Soil-Borne Pathogen Fusarium Oxysporum. Mol. Plant Microbe Interact..

[45] Zhalnina K., Louie K.B., Hao Z., Mansoori N., Da Rocha U.N., Shi S., Cho H., Karaoz U., Loqué D., Bowen B.P. (2018). Dynamic Root Exudate Chemistry and Microbial Substrate Preferences Drive Patterns in Rhizosphere Microbial Community Assembly. Nat. Microbiol..

[46] Bais H.P., Walker T.S., Schweizer H.P., Vivanco J.M. (2002). Root Specific Elicitation and Antimicrobial Activity of Rosmarinic Acid in Hairy Root Cultures of Ocimum Basilicum. Plant Physiol. Biochem..

[47] Zhang J., Subramanian S., Stacey G., Yu O. (2009). Flavones and Flavonols Play Distinct Critical Roles during Nodulation of Medicago Truncatula by *Sinorhizobium meliloti*. Plant J. Cell Mol. Biol..

[48] Lombardi N., Vitale S., Turrà D., Reverberi M., Fanelli C., Vinale F., Marra R., Ruocco M., Pascale A., D’Errico G. (2018). Root Exudates of Stressed Plants Stimulate and Attract Trichoderma Soil Fungi. Mol. Plant Microbe Interact..

[49] Oota M., Tsai A.Y.L., Aoki D., Matsushita Y., Toyoda S., Fukushima K., Saeki K., Toda K., Perfus-Barbeoch L., Favery B. (2020). Identification of Naturally Occurring Polyamines as Root-Knot Nematode Attractants. Mol. Plant.

[50] Gao X., Li T., Liu W., Zhang Y., Shang D., Gao Y., Qi Y., Qiu L. (2020). Enhancing the 1-Aminocyclopropane-1-Carboxylate Metabolic Rate of *Pseudomonas* Sp. UW4 Intensifies Chemotactic Rhizocompetence. Microorganisms.

[51] Tohidifar P., Plutz M.J., Ordal G.W., Rao C.V. (2019). The Mechanism of Bidirectional PH Taxis in *Bacillus subtilis*. J. Bacteriol..

[52] Xie Z., Ulrich L.E., Zhulin I.B., Alexandre G. (2010). PAS Domain Containing Chemoreceptor Couples Dynamic Changes in Metabolism with Chemotaxis. Proc. Natl. Acad. Sci. USA.

[53] Alexandre G. (2010). Coupling Metabolism and Chemotaxis-Dependent Behaviours by Energy Taxis Receptors. Microbiology.

[54] Taylor B.L., Zhulin I.B., Johnson M.S. (1999). Aerotaxis and Other Energy-Sensing Behavior in Bacteria. Annu. Rev. Microbiol..

[55] Rebbapragada A., Johnson M.S., Harding G.P., Zuccarelli A.J., Fletcher H.M., Zhulin I.B., Taylor B.L. (1997). The Aer Protein and the Serine Chemoreceptor Tsr Independently Sense Intracellular Energy Levels and Transduce Oxygen, Redox, and Energy Signals for Escherichia Coli Behavior. Proc. Natl. Acad. Sci. USA.

[56] Arrebola E., Cazorla F.M. (2020). Aer Receptors Influence the *Pseudomonas chlororaphis* PCL1606 Lifestyle. Front. Microbiol..

[57] Zhang W., Li X.G., Sun K., Tang M.J., Xu F.J., Zhang M., Dai C.C. (2020). Mycelial Network-Mediated Rhizobial Dispersal Enhances Legume Nodulation. ISME J..

[58] Muok A.R., Claessen D., Briegel A. (2021). Microbial Hitchhiking: How Streptomyces Spores Are Transported by Motile Soil Bacteria. ISME J..

[59] Parkinson J.S., Hazelbauer G.L., Falke J.J. (2015). Signaling and Sensory Adaptation in Escherichia Coli Chemoreceptors: 2015 Update. Trends Microbiol..

[60] Matilla M.A., Martín-Mora D., Krell T. (2020). The Use of Isothermal Titration Calorimetry to Unravel Chemotactic Signalling Mechanisms. Environ. Microbiol..

[61] Flack C.E., Parkinson J.S. (2018). A Zipped-Helix Cap Potentiates HAMP Domain Control of Chemoreceptor Signaling. Proc. Natl. Acad. Sci. USA.

[62] Sourjik V., Wingreen N.S. (2012). Responding to Chemical Gradients: Bacterial Chemotaxis. Curr. Opin. Cell Biol..

[63] Lacal J., García-Fontana C., Muñoz-Martínez F., Ramos J.L., Krell T. (2010). Sensing of Environmental Signals: Classification of Chemoreceptors According to the Size of Their Ligand Binding Regions. Environ. Microbiol..

[64] Matilla M.A., Krell T. (2017). Chemoreceptor-Based Signal Sensing. Curr. Opin. Biotechnol..

[65] Alexandre G., Greer-phillips S., Zhulin I.B. (2004). Ecological Role of Energy Taxis in Microorganisms. FEMS Microbiol. Rev..

[66] Krell T., Lacal J., Muñoz-Martínez F., Reyes-Darias J.A., Cadirci B.H., García-Fontana C., Ramos J.L. (2011). Diversity at Its Best: Bacterial Taxis. Environ. Microbiol..

[67] Fernandez M., Corral-Lugo A., Krell T. (2018). The Plant Compound Rosmarinic Acid Induces a Broad Quorum Sensing Response in *Pseudomonas aeruginosa* PAO1. Environ. Microbiol..

[68] Raina J.-B., Fernandez V., Lambert B., Stocker R., Seymour J.R. (2019). The Role of Microbial Motility and Chemotaxis in Symbiosis. Nat. Rev. Microbiol..

[69] Sampedro I., Parales R.E., Krell T., Hill J.E. (2015). *Pseudomonas* Chemotaxis. FEMS Microbiol. Rev..

[70] Kato J., Kim H.E., Takiguchi N., Kuroda A., Ohtake H. (2008). *Pseudomonas aeruginosa* as a Model Microorganism for Investigation of Chemotactic Behaviors in Ecosystem. J. Biosci. Bioeng..

[71] Fernández M., Matilla M.A., Ortega Á., Krell T. (2017). Metabolic Value Chemoattractants Are Preferentially Recognized at Broad Ligand Range Chemoreceptor of *Pseudomonas putida* KT2440. Front. Microbiol..

[72] Glekas G.D., Foster R.M., Cates J.R., Estrella J.A., Wawrzyniak M.J., Rao C.V., Ordal G.W. (2010). A PAS Domain Binds Asparagine in the Chemotaxis Receptor McpB in *Bacillus subtilis*. J. Biol. Chem..

[73] Zatakia H.M., Arapov T.D., Meier V.M., Scharf B.E. (2018). Cellular Stoichiometry of Methyl-Accepting Chemotaxis Proteins in *Sinorhizobium meliloti*. J. Bacteriol..

[74] Jiang N., Liu W., Li Y., Wu H., Zhang Z., Alexandre G., Elmerich C., Xie Z. (2016). A Chemotaxis Receptor Modulates Nodulation during the Azorhizobium Caulinodans-Sesbania Rostrata Symbiosis. Appl. Environ. Microbiol..

[75] López-Farfán D., Reyes-Darias J.A., Matilla M.A., Krell T. (2019). Concentration Dependent Effect of Plant Root Exudates on the Chemosensory Systems of *Pseudomonas putida* KT2440. Front. Microbiol..

[76] Feng H., Zhang N., Fu R., Liu Y., Krell T., Du W., Shao J., Shen Q., Zhang R. (2019). Recognition of Dominant Attractants by Key Chemoreceptors Mediates Recruitment of Plant Growth-Promoting Rhizobacteria. Environ. Microbiol..

[77] Zhang L., Li S., Liu X., Wang Z., Jiang M., Wang R., Xie L., Liu Q., Xie X., Shang D. (2020). Sensing of Autoinducer-2 by Functionally Distinct Receptors in Prokaryotes. Nat. Commun..

[78] Rico-Jiménez M., Muñoz-Martínez F., García-Fontana C., Fernandez M., Morel B., Ortega Á., Ramos J.L., Krell T. (2013). Paralogous Chemoreceptors Mediate Chemotaxis towards Protein Amino Acids and the Non-Protein Amino Acid Gamma-Aminobutyrate (GABA). Mol. Microbiol..

[79] Matilla M.A., Ortega Á., Krell T. (2021). The Role of Solute Binding Proteins in Signal Transduction. Comput. Struct. Biotechnol. J..

[80] Liu Y., Feng H., Fu R., Zhang N., Du W., Shen Q., Zhang R. (2020). Induced Root-Secreted D -Galactose Functions as a Chemoattractant and Enhances the Biofilm Formation of *Bacillus velezensis* SQR9 in an McpA-Dependent Manner. Appl. Microbiol. Biotechnol..

[81] Liu Y.C., Machuca M.A., Beckham S.A., Gunzburg M.J., Roujeinikova A. (2015). Structural Basis for Amino-Acid Recognition and Transmembrane Signalling by Tandem Per-Arnt-Sim (Tandem PAS) Chemoreceptor Sensory Domains. Acta Crystallogr. Sect. D Biol. Crystallogr..

[82] Nishiyama S.I., Takahashi Y., Yamamoto K., Suzuki D., Itoh Y., Sumita K., Uchida Y., Homma M., Imada K., Kawagishi I. (2016). Identification of a Vibrio Cholerae Chemoreceptor That Senses Taurine and Amino Acids as Attractants. Sci. Rep..

[83] Machuca M.A., Johnson K.S., Liu Y.C., Steer D.L., Ottemann K.M., Roujeinikova A. (2017). Helicobacter Pylori Chemoreceptor TlpC Mediates Chemotaxis to Lactate. Sci. Rep..

[84] Gavira J.A., Gumerov V.M., Rico-Jiménez M., Petukh M., Upadhyay A.A., Ortega A., Matilla M.A., Zhulin I.B., Krell T. (2020). How Bacterial Chemoreceptors Evolve Novel Ligand Specificities. mBio.

[85] Olson M.S., Ford R.M., Smith J.A., Fernandez E.J. (2004). Quantification of Bacterial Chemotaxis in Porous Media Using Magnetic Resonance Imaging. Environ. Sci. Technol..

[86] Armitage J.P., Gallagher A., Johnston A.W.B. (1988). Comparison of the Chemotactic Behaviour of Rhizobium Leguminosarum with and without the Nodulation Plasmid. Mol. Microbiol..

[87] Ni B., Huang Z., Fan Z., Jiang C.Y., Liu S.J. (2013). Comamonas Testosteroni Uses a Chemoreceptor for Tricarboxylic Acid Cycle Intermediates to Trigger Chemotactic Responses towards Aromatic Compounds. Mol. Microbiol..

[88] Hida A., Oku S., Kawasaki T., Nakashimada Y., Tajima T., Kato J. (2015). Identification of the McpA and McpM Genes, Encoding Methyl-Accepting Proteins Involved in Amino Acid and l-Malate Chemotaxis, and Involvement of McpM-Mediated Chemotaxis in Plant Infection by *Ralstonia pseudosolanacearum* (Formerly *Ralstonia solanacearum* Phylotypes I and III). Appl. Environ. Microbiol..

[89] Yost C.K., Rochepeau P., Hynes M.F. (1998). Rhizobium Leguminosarum Contains a Group of Genes That Appear to Code for Methyl-Accepting Chemotaxis Proteins. Microbiology.

